# Additive Manufactured Large Zr-Based Bulk Metallic Glass Composites with Desired Deformation Ability and Corrosion Resistance

**DOI:** 10.3390/ma13030597

**Published:** 2020-01-28

**Authors:** Yu Luo, Leilei Xing, Yidong Jiang, Ruiwen Li, Chao Lu, Rongguang Zeng, Jinru Luo, Pengcheng Zhang, Wei Liu

**Affiliations:** 1 Institute of Materials, China Academy of Engineering Physics, P.O.Box 9071, Jiangyou 621907, Sichuan, China; 15181680856@163.com (Y.L.); jiang219@mail.ustc.edu.cn (Y.J.); ruiwenli@163.com (R.L.); luchaooo@gmail.com (C.L.); zengrongguang@caep.cn (R.Z.); jinruluo@163.com (J.L.); 2 School of Materials Science and Engineering, Tsinghua University, Beijing 100084, China; xingll16@mails.tsinghua.edu.cn

**Keywords:** Zr_60_Fe_10_Cu_20_Al_10_ bulk metallic glass, selective laser melting, laser parameters, nano-crystals, mechanical property, corrosion resistance

## Abstract

Zr-based bulk metallic glasses have been attracting tremendous interest of researchers because of their unique combination of mechanical and chemical properties. However, their application is limited as large-scale production is difficult due to the limitation of cooling rate. Recently, additive manufacturing technology has been proposed as a new solution for fabricating bulk metallic glasses without size limitation. In this study, selective laser melting technology was used to prepare Zr_60_Fe_10_Cu_20_Al_10_ bulk metallic glass. The laser parameters for fabricating full dense amorphous specimens were investigated. The mechanical and corrosion resistance properties of the prepared samples were measured by micro-compression and electrochemical corrosion testing, respectively. Lastly, Zr_60_Fe_10_Cu_20_Al_10_ bulk metallic glass (BMG) with dispersed nano-crystals was made, and good deformation ability was revealed during micro-compression test. The corrosion resistance decreased a bit due to the crystalline phases. The results provide a promising route for manufacturing large and complex bulk metallic glasses with better mechanical property and acceptable corrosion resistance.

## 1. Introduction

In past decades, a variety of Zr-based bulk metallic glasses (BMGs) have been developed because of their high glass forming ability (GFA) [1]. These Zr-based BMGs show very attractive properties such as ultrahigh strength with a low Young’s modulus and high corrosion resistance [2,3,4]. These superior properties make Zr-based BMGs hold promises for application in various fields. Therefore, searching for a suitable technique for fabricating BMG parts with high efficiency and low cost has become an important issue for promoting their practical application.

Compared with crystalline solids, BMGs are alloys with disordered atomic arrangements. It is known that appropriate compositional selection and rapid cooling rate are keys to make these BMGs. For many years, water-cooling suction casting and injection casting have been used for most BMGs’ fabrication [5,6]. By alloy designing, several BMG systems were produced over a critical diameter (D_c_) of 20 mm owing to the improved GFA [7,8,9,10]. It is known that the GFA is highly related to thermal stability, and by adding the minor elements the thermal stability of the BMG can be enhanced; thus, the GFA can be enhanced in the same time [11]. However, the maximum critical diameter for BMG formation is about 100 mm [12], beyond which it is difficult to cast BMGs with fully amorphous structure by using the conventional casting technique. Therefore, works on improving GFA to obtain BMGs with larger critical diameters have become more difficult.

Most of the BMGs show a brittle character due to lack of dislocations, which exist in their crystalline counter parts, and that leads to the result that the BMGs are hard to deform into complex geometries [13]. These two issues make it hard to build BMGs with large geometric dimensioning and desired shapes for further application. 

Selective laser melting (SLM) is one of the additive manufacturing technologies. In the SLM procedure, a three dimensional part is pre-designed by computer aided design (CAD) and is fabricated layer by layer on a powder bed [14,15]. This makes the fabrication of complex geometry parts quite easy by using SLM technology. Due to the short interaction time between laser beam and powders, rapid melting and subsequent solidification occur, leading to very high heating and cooling rates (10^3^–10^8^ K/s) [16,17]. Such high cooling rates meet the requirement for forming BMGs. Usually, the diameters of laser spots of the SLM equipment are smaller than those of other additive manufacturing technologies. It is interesting to notice that the fabrication of an SLM part is to overlay the procedures of small melting pools generated by laser spots in micro-diameter, which makes the SLM method bypass the D_c_ problem encountered by other conventional methods. Some research has been made on BMG additive manufacturing in recent years; Fe-, Zr-, and Al-based amorphous alloys were fabricated, and nano-crystals were found in the heat-affected zone (HAZ) [18,19,20,21,22]. 

It has been reported that the fundamental unit of plastic deformation of BMGs is the shear transformation zone (STZ) [23,24], and shear band plays an important role during deformation process. Studies have suggested that as the volume fraction of non-crystalline phase increases, the plasticity increases [25,26,27]. The basic idea is to block the propagation of the shear bands during the plastic deformation. The research shows that during the SLM process, BMGs with nano-crystals are formed by thermal recycling or stress [21,28]; however, when the volume fraction of nano-crystal is lower than 20%, the deformation ability decreases with the reduction of volume fraction [29]. This enlightens us to enhance the ductility of BMGs by introducing nano-crystals within the amorphous matrix with a volume fraction under 20% by SLM technology. The corrosion resistance can be maintained by controlling the volume fraction of nano-crystalline phase.

In this study, bulk metallic glass composites with nominal composition of Zr_60_Fe_10_Cu_20_Al_10_ (in at%) with a critical cast diameter of 10 mm were fabricated by SLM equipment with pulse laser beam using gas atomized amorphous powder [30]. The mechanical and corrosion resistance properties of the prepared samples were investigated by micro-compression test and electrochemical corrosion measurement. 

## 2. Experiment

### 2.1. Materials Preparation

Zr-based metallic glass powder with a composition of Zr_60_Fe_10_Cu_20_Al_10_ (in at%) was used in this study. To avoid adding superfluous elements, the master ingot with a nominal composition of Zr_60_Fe_10_Cu_20_Al_10_ (in at%) was firstly prepared by using vacuum suspension melting high-purity ingots of Zr (>99.9 wt%), Fe (>99.99 wt%), Cu (>99.99 wt%), and Al (99.99 wt%) four times to ensure the chemical homogeneity. Then, the master ingot was used to prepare powder through gas atomization. The powder with a diameter of 15–53 μm was selected through a sieve to ensure that the powder size was suitable for the SLM equipment and to make the powder have sufficient flow ability during the laser processing. Then, the powder was heated in a vacuum drying oven. The primary structure of the powder was evaluated by X-ray diffraction (XRD) using Cu-Kα radiation. The broad diffraction peak in the pattern of the powder indicated a fully amorphous character.

Cubic specimens with dimensions of 8 × 8 × 4 mm^3^ and cylindrical SLM specimens with dimensions of Φ4× 30 mm^3^ were prepared on Zr substrates by a Renishaw AM250 SLM device equipped with a SPI(Britain) redPOWER 200 W ytterbium fiber laser (operating at 1071 nm) and focused at a 75 μm spot diameter. Selected parameters include laser power (P) ranging between 100 W and 200 W, exposure time (t) between 20 μs and 100 μs with a hatch space of 80 μm, and point distance of 70 μm. Cylindrical specimens with a diameter of 4 mm and a height of 30 mm were prepared with parameters of P = 200 W, t = 40, 50, 60, 70 μs. Before the laser processing, a low oxygen environment (<100 ppm) was obtained using high purity argon after the molecular pump finished its job. The as-cast Zr_60_Fe_10_Cu_20_Al_10_ rod with a diameter of 4 mm was fabricated under Ar atmosphere by suction casting in a water-cooled copper mold as a comparison sample. The structure of the specimens was measured by using laser scanning confocal microscope (LSCM), differential scanning calorimetry (DSC), XRD and high resolution transmission electron microscope (HRTEM).

### 2.2. Mechanical Properties Testing 

The micro hardness of the as-cast rod and cylindrical SLM specimens with parameters of P = 200 W, t = 40, 50, 60, 70 μs were tested by a Vickers hardness gauge. The specimens used during micro-compression were cut from the as-casted rod, and the SLM specimens had parameters of P = 200 W, t = 40, 50, 60 μs. Then, the specimens were mechanically polished with an automated lapping machine. After mechanical polishing, these four specimens were then electro-polished to ensure no residual damage was left.

The micro-compression specimens were prepared by using a focused ion beam system with 30 kV Ga^+^ ions. In this process, the specimens’ surfaces were oriented normal to the ion beam column to ensure that the flat indenter tip (20 nm in diameter) did not contact any other surface of the micro-compression rod and make it easier for the microscope of the nano-indenter to find the rod. An annular milling pattern with diameter of 30 nm was firstly selected. Then, several smaller concentric annular patterns were used as well as smaller beam currents. Lastly, a micro-rod with dimensions of 3 ± 0.5 μm diameter and 5 ± 0.5 μm height was prepared.

The fabricated micro-pillars were compressed by a nano-indenter with a flat indenter tip in a dynamic contact mode. The pillars were compressed under an increasing load (maximum = 10 mN) at a constant rate and then unloaded at a constant rate. The displacement of the pillars was measured during the compression.

### 2.3. Electrochemical Corrosion Testing

The specimens used in this test were also cut from the as-casted rod and the SLM specimens with parameters of P = 200 W, t = 40, 50, 60, and 70 μs, then fixed in epoxy resin and the untested side of specimens were linked with copper wires. Then, these specimens were mechanically polished by an automated lapping machine then cleaned by sonication in anhydrous alcohol for 600 s and sequentially dried by Ar flow. The effective area was 12.56 mm^2^ each. A three-electrode system was used, including a Zr_60_Fe_10_Cu_20_Al_10_ specimen as the working electrode, a 4 cm^2^ Pt net as the counter electrode, and a saturated calomel electrode as the reference electrode. The electrolyte was NaCl solution (3.5 wt%), and the electrolytic cell was a commercial flat electrolytic cell.

The three-electrode system was steeped in the NaCl solution (3.5 wt%) for 600 s to stabilize the open-circuit potential (OPC). Then, potentiodynamic polarization was conducted, where the sweep potential was ±150 mV versus OPC and the sweep rate was 0.5 mV/s.

## 3. Results

### 3.1. Selective Laser Melting Processing

**Figure 1:**
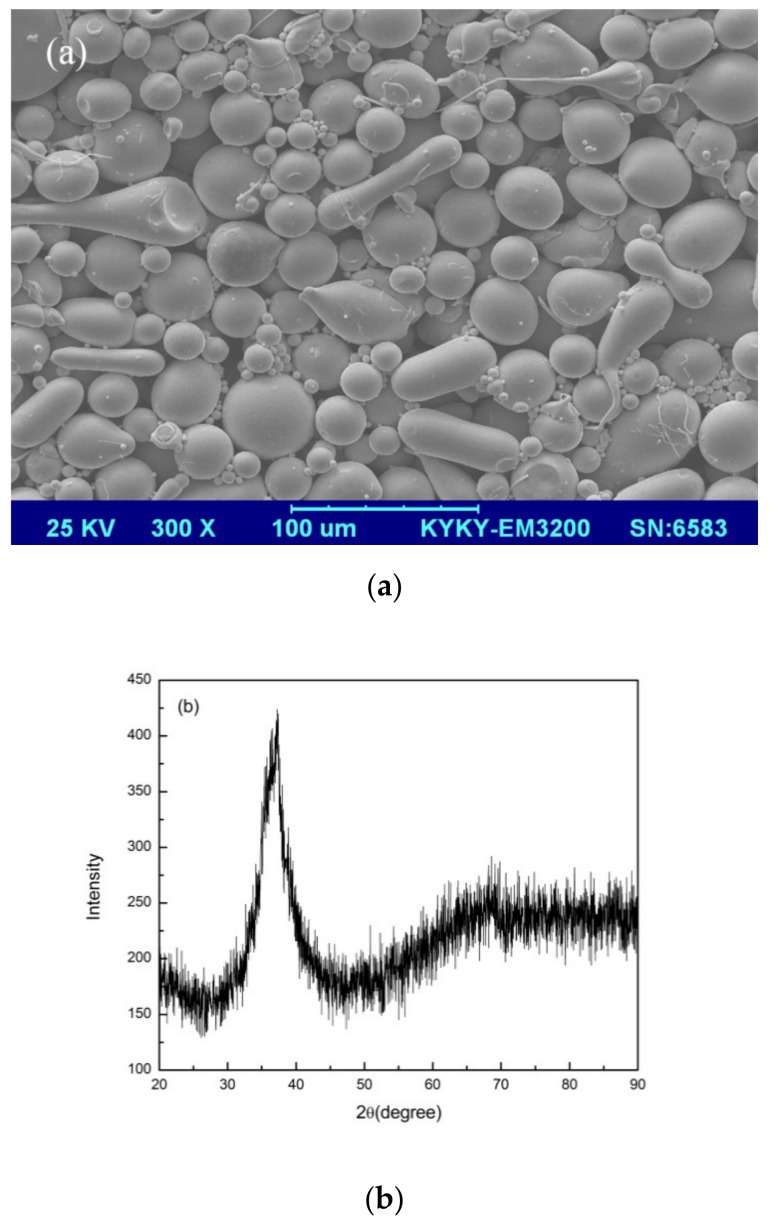
(**a**) SEM image and (**b**) XRD pattern of the Zr_60_Fe_10_Cu_20_Al_10_ metallic glass powder.

The XRD pattern of gas-atomized Zr_60_Fe_10_Cu_20_Al_10_ (in at%) powders with the size in the range of 15–53 μm is shown in Figure 1a. A broad and diffuse halo pattern can be observed, suggesting fully amorphous structure of the powders. Figure 1b shows the geometrical morphologies of these as-fabricated powders, which indicate an acceptable flow ability of the amorphous powders. 

**Figure 2:**
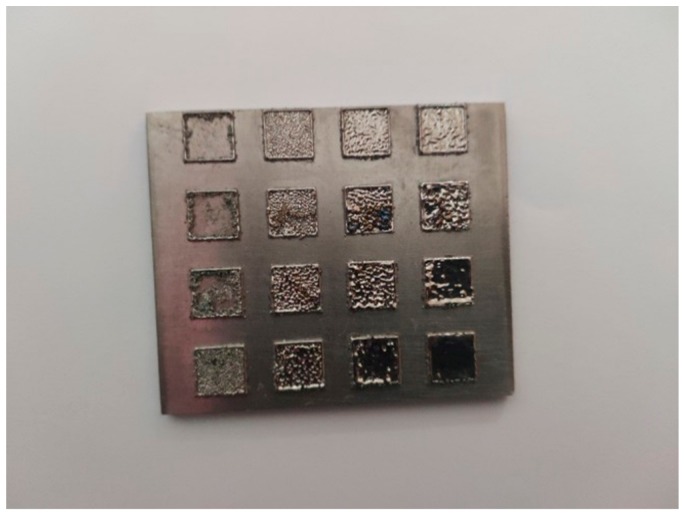
Results of primary study on the laser parameters range.

**Figure 3:**
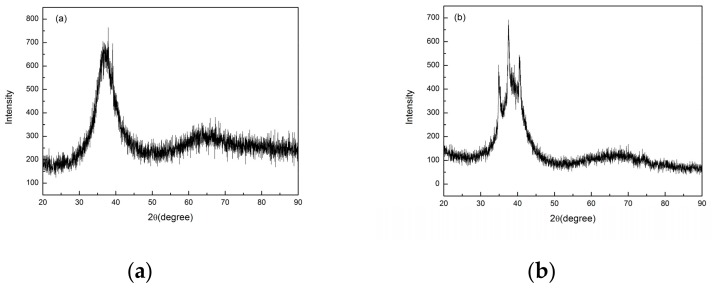
Two typical XRD patterns of the of SLM specimens, (**a**) fully amorphous, (**b**) amorphous with partial crystallinity.

**Figure 4:**
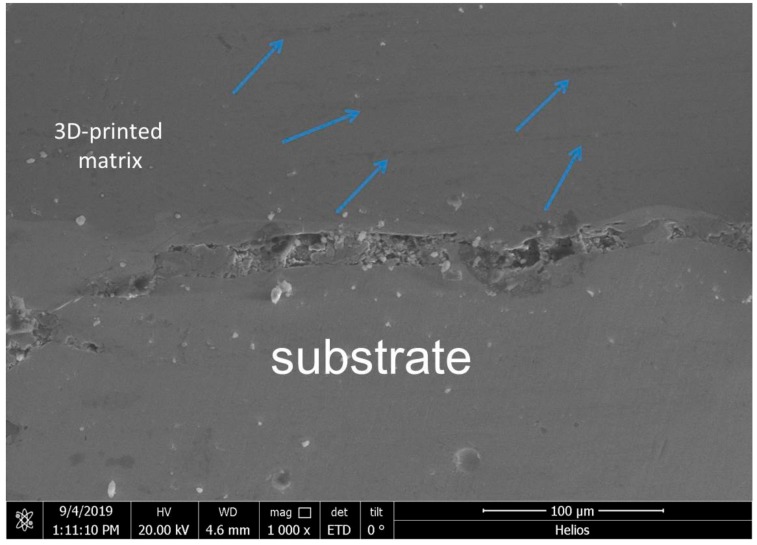
Distribution of crystalline phases in 3D-printed matrix.

**Figure 5:**
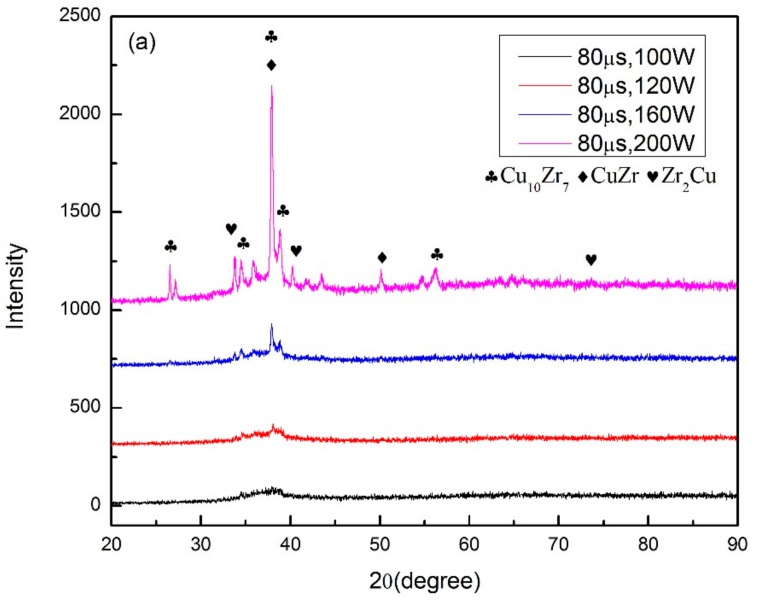

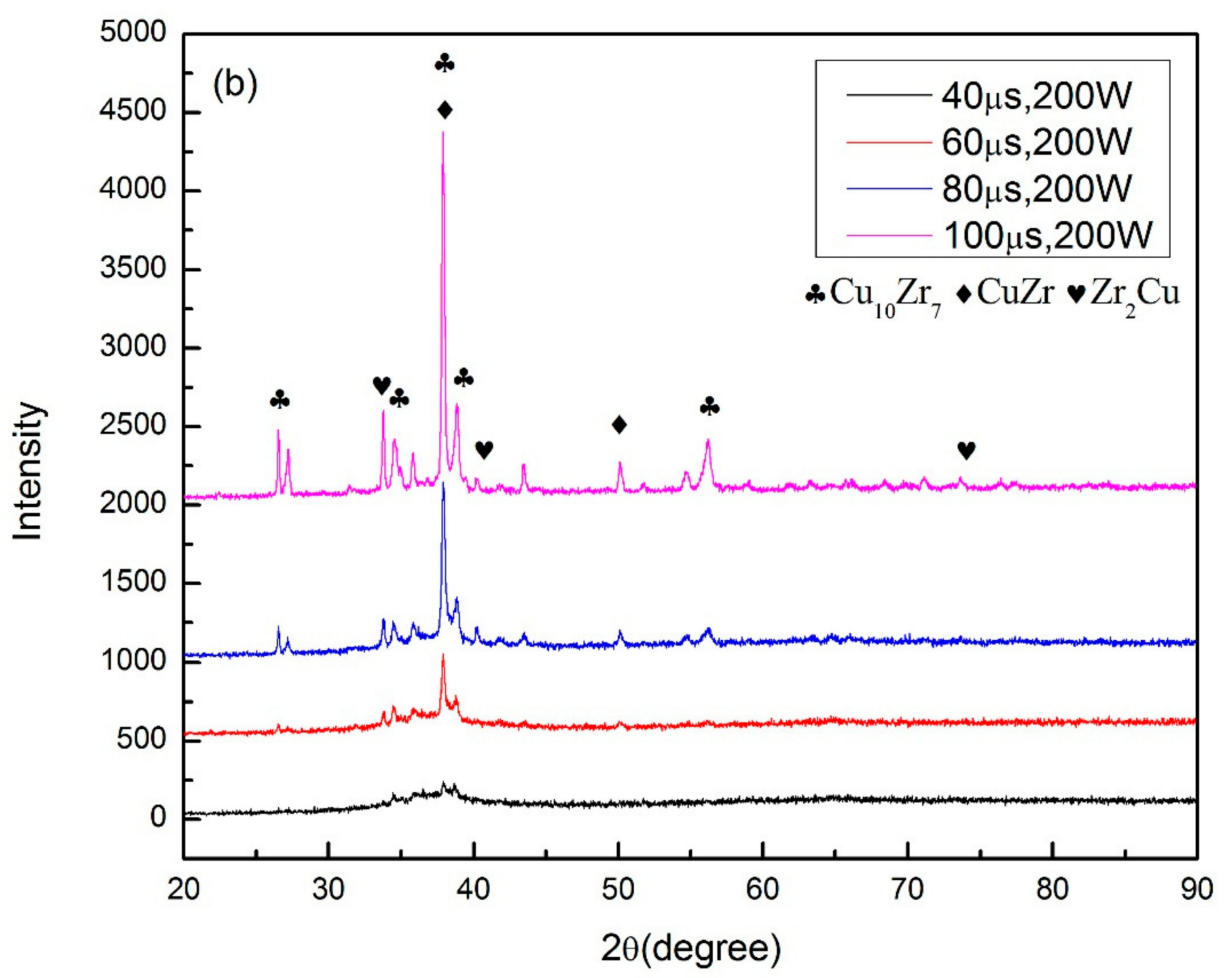
XRD patterns of (**a**) different laser power with same exposure time; (**b**) different exposure time with same laser power.

**Figure 6:**
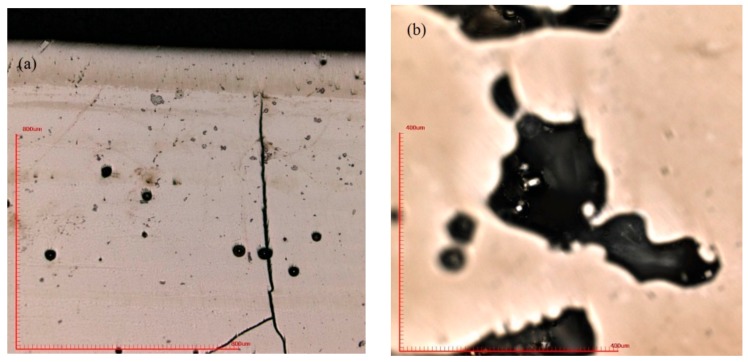
(**a**) Cracks and macro-pores revealed in the SLM matrix (P = 200 W, t = 60 μs); (**b**) partially melted powder in the macro-pore (P = 120 W, t = 60 μs).

**Figure 7:**
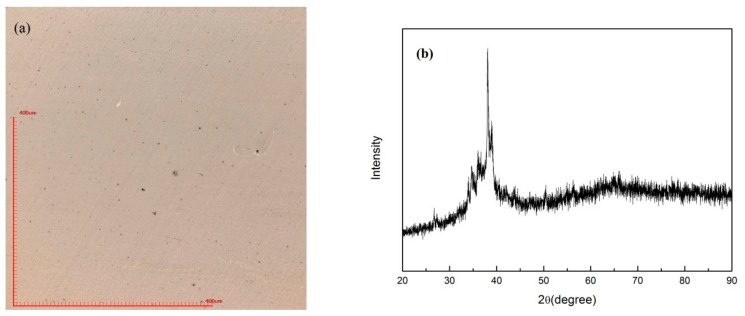
SLM specimen (P = 160 W, t = 60 μs) fabricated by re-melt scanning strategy. (**a**) optical microscope image; (**b**) XRD pattern.

**Figure 8:**
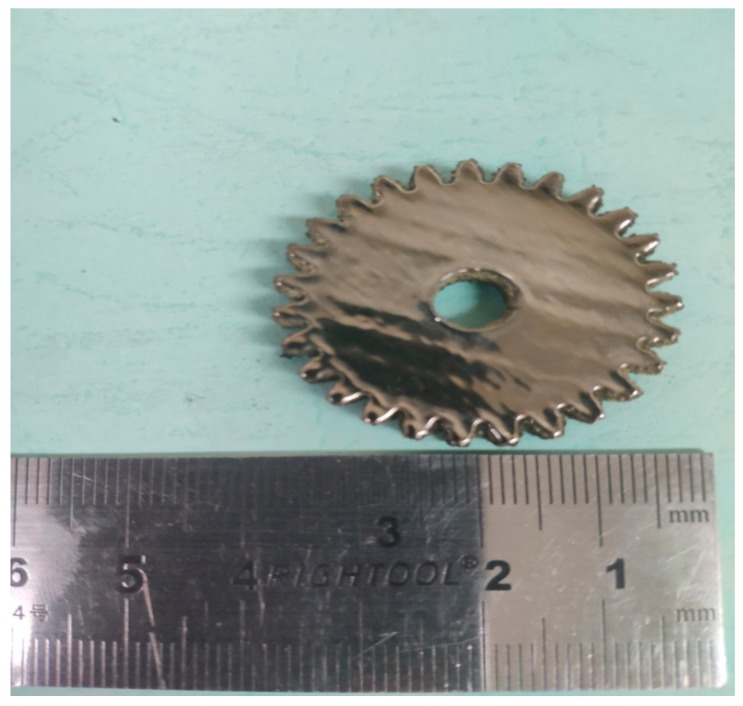
SLM-fabricated Zr_60_Fe_10_Cu_20_Al_10_ BMG part (diameter 35 mm and height 5 mm).

In order to find out the appropriate parameter range to prepare 3-dimentional parts, a preliminary work was done before fabricating the Zr_60_Fe_10_Cu_20_Al_10_ BMG. A series of parameters were set as shown in Table 1. Ten layers were set to melt in the program. According to the results shown in Figure 2, when laser power P ≤ 160 W, the parameters with exposure time of 20 μs cannot be selected to fabricate the 3-dimensional parts, for the insufficient laser energies caused by short exposure time. Then, another series of parameters was selected as shown in Table 2. The structures of the specimens fabricated by SLM were determined by XRD using the cross-section of the samples. Two typical patterns of (i) nearly full amorphous structure and (ii) partially crystallized are shown in Figure 3. Figure 4 shows the cross section of the 3D-printed specimen. It can be seen that the crystalline phases were distributed in the HAZ along the scanning direction. In this investigation, parameter study was mainly focused on the laser power and exposure time. By comparing the patterns acquired from parameters in different laser powers with the same exposure time and in different exposure times with the same laser power (Figure 5), it is indicated that the volume fraction of the amorphous phase decreases with the increase of the laser power or exposure time. However, macro-pores and cracks widely exist in specimens with lower laser energies or shorter exposure time (Figure 6) [31]. This suggests the laser energies were insufficient for making fully dense Zr_60_Fe_10_Cu_20_Al_10_ BMGs by using these parameters. To fabricate crack- and pore-free BMG specimens, the scan strategy was changed. The selected laser parameters were still the same as in Table 2, and the re-melting parameters were all P = 100 W and t = 30 μs. Under the new SLM process, each layer was melted twice by laser beam. The results show that nearly fully dense specimens of Zr_60_Fe_10_Cu_20_Al_10_ BMGs containing a small number of crystalline phases can be formed under these conditions (Figure 7); however, a few micro-pores can still be observed in the as-printed matrix (Figure 7a). A simple gear was built with the parameters of P = 160 W and t = 60 μs and was melted twice, exhibiting a probable future of building BMGs in large and complex geometry (Figure 8).

**Figure 9:**
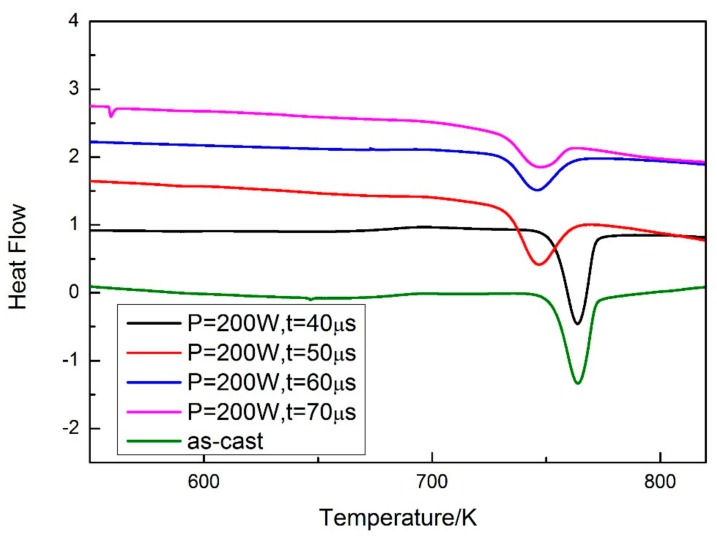
DSC curves of as-cast and SLM specimens.

A DSC test was carried out for evaluating the volume fraction of the amorphous phase in the SLM specimens. The specimens were cut from the as-cast rod and cylindrical SLM specimens, and the specimens used in the DSC test were thinned to ensure accuracy. The cast rod was used as a standard specimen, and the volume fraction of the amorphous phase was calculated by comparing the crystallization enthalpy of the SLM specimens with the standard specimen. The results of the DSC test are shown in Figure 9. Both the crystallization enthalpy and volume fraction of the amorphous phase can be calculated (Table 3). 

### 3.2. Mechanical Properties

**Figure 10:**
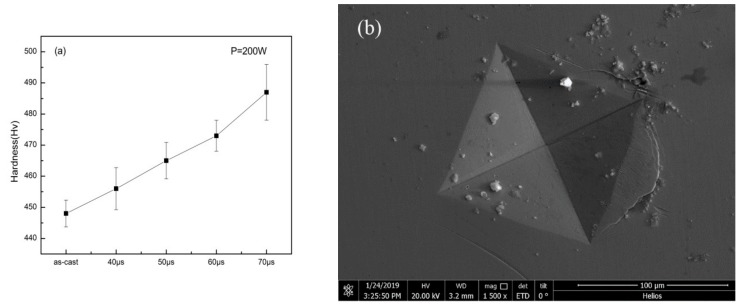
(**a**) Vickers hardness results of as-cast specimen and specimens with different exposure times; (**b**) indentation on specimen with parameters of P = 200 W, t = 40 μs.

Figure 10a shows the result of micro-hardness measurement. It can be seen that as the exposure time increased, the hardness of the specimens increased compared to the as-cast specimen. It is interesting to notice that, although Zr-based BMG is generally considered as a brittle material, the crack caused by the hardness gauge revealed in Figure 10b suggests the ductility that the specimen may have.

**Figure 11:**
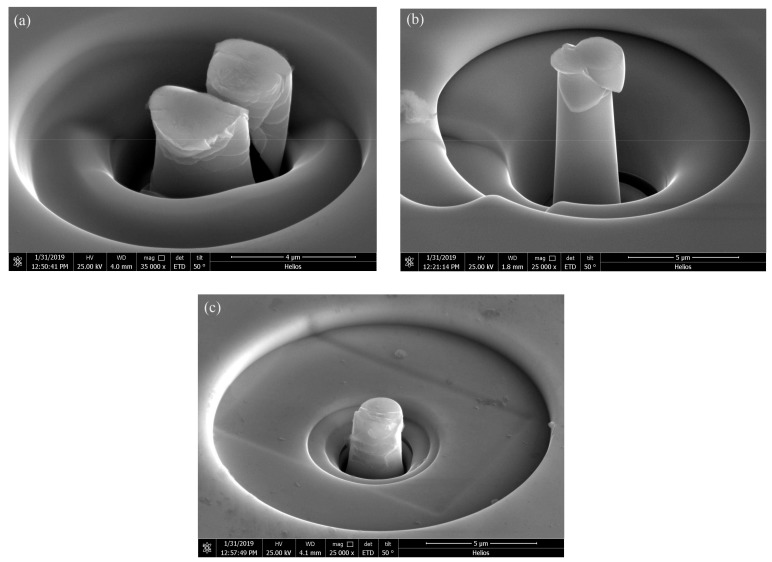
The morphologies of the compressed pillars, (**a**) as-cast, (**b**) parameters of P = 200 w, t = 40 μs, (**c**) parameters of P = 200 W, t = 50 μs.

The morphologies of the compressed pillars are shown in Figure 11. In Figure 11a, the pillar fractured after the compression. Figure 11b shows that the pillar deformed by several shear bands generated after the pillar yields. Figure 11c shows the morphology of the compressed pillar prepared with the parameters of P = 200 w and t = 50 μs. There is no clear major shear band revealed in the image.

### 3.3. Corrosion Resistance Measurement

**Figure 12:**
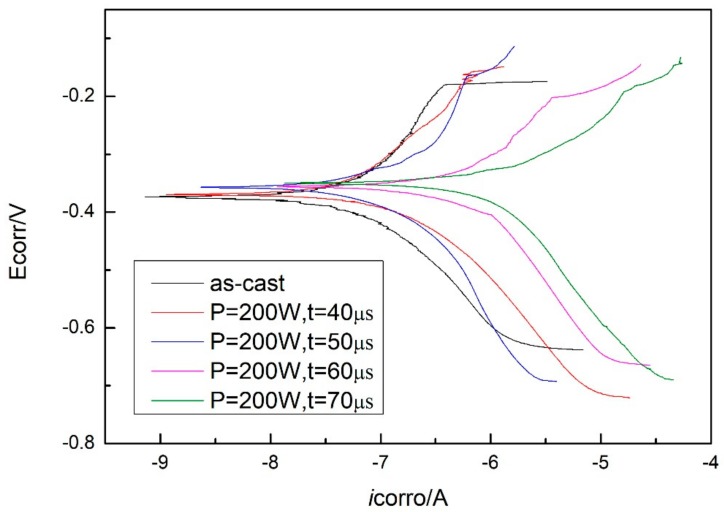
Tafel plots of as-cast specimen and SLM specimens with parameters of P = 200 W, t = 40, 50, 60, and 70 μs.

The potentiodynamic polarization curves of the as-cast Zr-based BMG and SLM specimens (with parameters of P = 200 W and t = 40, 50, 60, and 70 μs) tested in simulated seawater are shown in Figure 12. A number of corrosion-related parameters including the corrosion potential (Ecorr), the corrosion current density (icorr), and the corrosion penetration rate (CPR) were identified and are summarized in Table 4. The corrosion penetration rate (CPR, μm/year) is calculated by using Faraday’s law [32]:

(1) $$\mathrm{CPR}=0.327\left(Mi_{\mathrm{corr}}\right)/m\rho$$

where *M* (g/mol), m, and *ρ* (g/cm3) are the atom-fraction-weighted values of atomic weight, ion valence, and density of the alloy elements, respectively, and icorr (mA/m2) is the corrosion current density. The as-cast Zr-based BMG exhibits lower corrosion rate, and the corrosion rates of SLM specimens are increased with the exposure time. 

## 4. Discussion

### 4.1. Laser Parameter Study

The SLM procedure can be considered as welding. The cooling process can be described by the equations below [33],

(2) $$T_{p}-T_{0}=\left(\frac{2}{\pi e}\right)\frac{q/v}{\rho cr^{2}}$$

(3) $$∆t={\frac{\left(q/v\right)}{4\pi \lambda \rho cd^{2}\theta }}^{2}$$

where *T_p_* is the peak temperature, *T*_0_ is the initial temperature, *ρc* is the specific heat per unit volume, v is the welding velocity, *q* the arc power, ∆t is the cooling time, *λ* is the thermal conductivity, and *θ* is a function related to *T*_0_ and *T*_p_. It can be inferred from the equations above that the cooling rate is highly dependent on the welding velocity and energy input. With a higher welding velocity or lower energy input, the cooling rate can be increased. In comparison to the welding procedure, lower laser power and shorter expose time imply a slower cooling rate during the SLM process, and this is proven by the XRD results measured from the cross-section specimens as shown in Figure 5. The three types of structures that represent (i) nearly fully amorphous, (ii) partial crystallized are indicated in colors of green and yellow in Table 2. It is important to notice that while the cooling rate of the SLM procedure is much larger than the critical cooling rate for the formation of Zr_60_Fe_10_Cu_20_Al_10_ BMGs, crystalline phases still exist in the specimens with higher laser power or longer expose time. It is mainly due to the heat accumulation in the heat-affected zone (HAZ), which can be seen in Figure 4, and a similar result was also reported by other researchers [34].

During the SLM procedure, a large thermal gradient exists due to the rapid melting and solidification. Thus, high residual stress is maintained in the as-fabricated three-dimensional part, which induces cracks in the matrix [35,36,37]. Micro pores were formed in the specimens mainly due to the insufficient laser energy. It is reported by the literature that laser re-scanning can reduce the residual stress effectively [38], and in this case it is a good way to increase the laser energy that the powders absorb. Thus, it is clear that after a laser re-melting process on the melted surface, most of the cracks and macro-pores were eliminated by decreasing the residual stress and increasing the laser energy input. 

### 4.2. Hardness and Micro-Compression 

In this work, the hardness of the specimens indeed increased with increasing exposure time because of the crystalline phases reinforcement. Although most of the cracks and pores were eliminated through parameter adjustment, some micro-pores still existed in the matrix (Figure 7a). It can be speculated that the compressive strength of the as-printed specimens cannot match the as-cast one. The deformation of BMGs is usually mediated by the sliding of shear bands. The dispersed nano-crystalline phases are supposed to prevent the pillar from cracking in one major shear band, which makes the SLM specimen harder to deform compared with the as-cast specimen.

**Figure 13:**
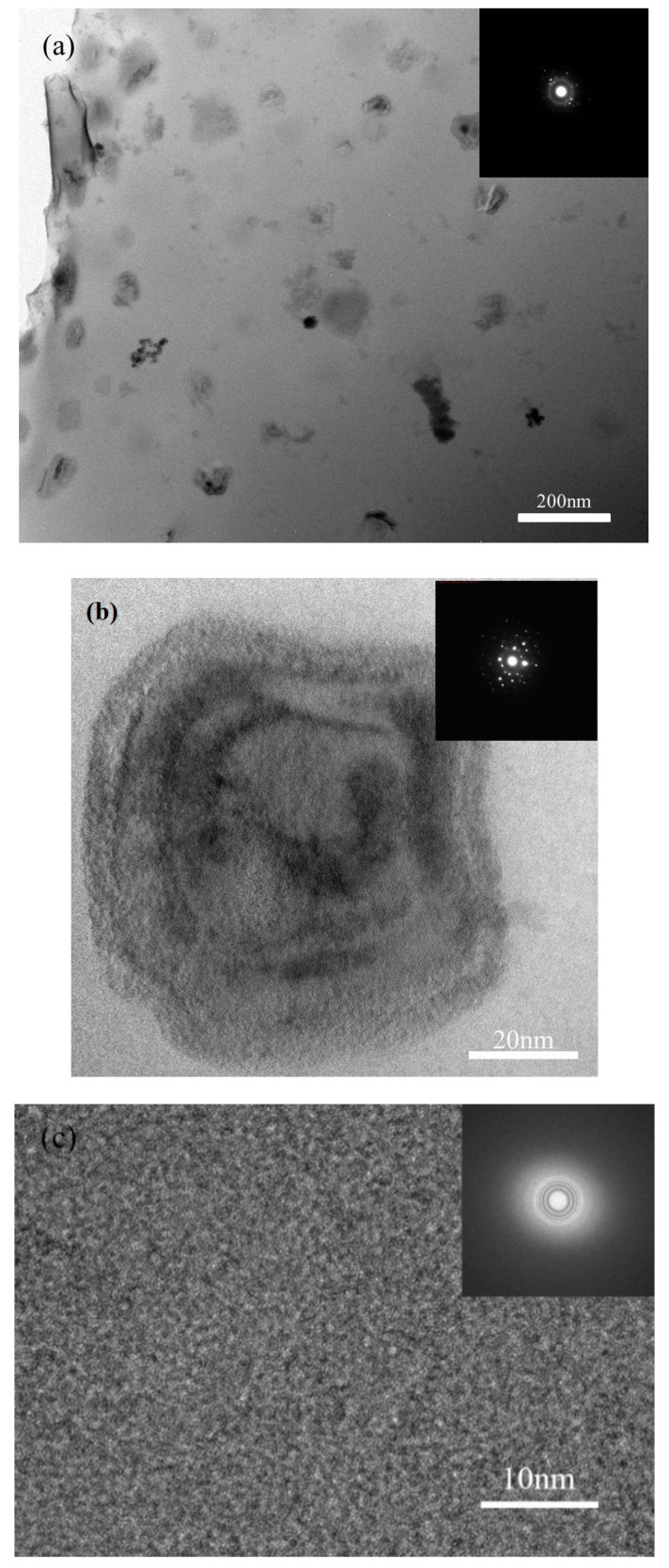
TEM images of SLM specimen with parameters of P = 200 W and t = 40 μs, (**a**) distribution of the nano-crystalline phases in amorphous matrix, (**b**) HRTEM image of selected nano-crystalline phase, (**c**) HRTEM image of the matrix.

**Figure 14:**
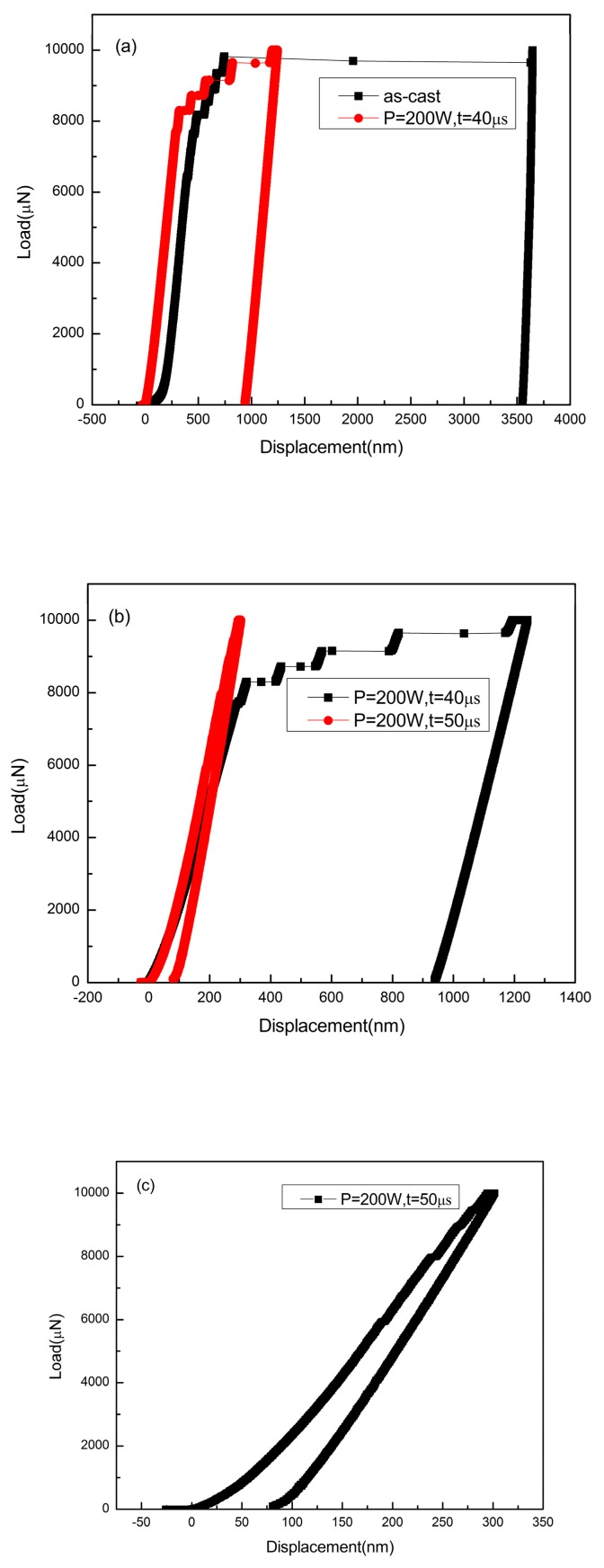
Load–displacement curves of micro-compression test: (**a**) as-cast specimen and the SLM specimen with parameters of P = 200 W and t = 40 μs; (**b**) SLM specimen with parameters of P = 200 W and t = 40 μs and P = 200 W and t = 50 μs; (**c**) SLM specimen with parameters of P = 200 W and t = 50 μs.

It is reported that heat accumulation or mechanical stress during the laser additive manufacturing procedure probably causes the nano-crystal nucleation [28,34]. We also found some nano-crystals in the SLM specimens by TEM (Figure 13). The distribution of the nano-crystalline phases is shown in Figure 13a, and the grain sizes are in diameter of 50–100 nm. These nano-grains might affect the results of the micro-compression test in some ways. The Focus Ion Beam (FIB) machined specimens usually have tapered geometry with smaller diameter on the top surface, where the stress concentration is easily generated. During micro-compression, the deformation starts from the top of the sample. As a result, the shear band of the SLM BMG of fully amorphous structure formed in the top of the pillar as shown in Figure 11a. However, Figure 11b,c reveals different deformation behavior. In Figure 11b, several shear bands appear instead of one major shear band, and the pillar is fractured in the direction of the shear bands. In Figure 11c, primary shear bands can be seen in the pillar from the top to the bottom. Assuming that the pillar deformed homogeneously during the elastic deformation, the dispersed nano-crystalline phases can be the stress concentration sites [37,39], thus making the shear bands distribute more homogeneously all around the pillar. The nano-crystalline phases shown in Figure 13a are in quite small diameters, which can be treated as ideal crystal; thus, these grains exhibit great hardness. The shear band was reported to generate at the interface of the grain and amorphous matrix during compression, and also the homogeneous distributed nano-crystals, which embedded in the matrix, may act as obstacles to branch the primary shear band into multiple shear bands and result in improving the ductility [40,41]. That makes the SLM pillars more deformable than the cast ones. From the DSC results, the crystal volume fraction of specimens P = 200 W, t = 40, 50 μs are 4.05 and 18.7%, respectively (Table 3). The displacement of specimens as-cast, P = 200 W, t = 40, 50 μs are 744, 921, 83 nm (Figure 14), and the engineering strains are 14.88%, 18.42%, and 1.66%, respectively. By adding 4.05% nano-crystals, the deformation ability of the Zr_60_Fe_10_Cu_20_Al_10_ pillar was enhanced. However, the strain of specimen P = 200 W and t = 50 μs is only 1.66%, which is mainly because the maximum force of the indenter is 10 mN and the hardness of the specimen is largely enhanced by the nano-crystals (18.7%). By comparing the results shown in Figure 14, the hardness of the pillars can be increased by increasing of the crystalline phases; thus, this specimen has not reached its compression limit. It is important to notice that although the strains of these specimens are larger than BMGs with similar composition in macro compression test, it does not mean that these two kinds of results can be compared at the same time. For compression test, more ductile character can be revealed in micro-scale. In our present work, the deformation ability of the pillar is higher than the cast one.

Figure 14a compares the load–displacement curves of the as-cast specimen and the SLM specimen with the parameters of P = 200 W and t = 40 μs. It can be seen that in the same loading conditions, the SLM specimen was harder to deform and had better deformability. Figure 14b shows the comparison between two SLM specimens, in which the specimen with the parameters P = 200 W and t = 50 μs shows a harder character at higher crystalline volume fraction. No fracture was found in this pillar after the maximum compression. However, Figure 14b,c shows that the shear bands occurred instantly, implying that the strain does not take place in a gradual and continuous fashion but in the form of bursts. Compared with the deformation behavior shown in Figure 14a, the multiple shear bands observed in Figure 10b,c indicate that the SLM Zr-based BMG with nano-crystalline phases may also have better deformability than the cast ones as shown by the pillars.

### 4.3. Corrosion Behavior

Bulk metallic glasses (BMGs) with amorphous structure often exhibit corrosion resistance one to two orders of magnitude higher than polycrystalline alloys with the same composition, which can be explained in terms of lacking structural defects like dislocations or grain boundaries [32,42]. A variety of corrosion resistant Zr-based BMGs have been developed, and it has been indicated that the corrosion resistance also depends on the two factors mentioned above [43,44]. Thus, the corrosion rates of SLM specimens increasing with the exposure time can be attributed to the decrease in amorphous content caused by laser thermal effect.

The CPRs for the Zr_60_Fe_10_Cu_20_Al_10_ alloy SLM specimens were determined to be 0.11 μm/year to 2.09 μm/year, which are far superior to those of the traditional AISI 316 (12.6 μm/year [45]) stainless steels, demonstrating the potential of this alloy as a viable engineering alloy for anti-corrosive applications.

## 5. Conclusions

In summary, it is feasible to prepare complex shaped parts of amorphous alloy with the SLM method larger than the traditional methods with the right parameters to increase cooling rate, and it is better to use lower laser power and higher expose time. Micro-pores and cracks can be reduced by re-melting method. The size of the SLM BMG part can be larger than that of casting. In this work, a BMG part of diameter = 35 mm and height = 5 mm was fabricated, which is much larger than casting D_c_ (10 mm). By introducing an appropriate number of crystalline particles into the amorphous matrix, the deformation ability of the SLM pillar P = 200 W and t = 40 μs can be effectively improved from 14.88% to 18.42% during micro-compression, which indicates that the SLM BMG may also acquire such character in the same way. The corrosion resistance of the amorphous alloy itself can be maintained at the same time. 

## Figures and Tables

**Table 1:** Summary of the selective laser melting (SLM) parameters in range study.

Laser Parameters			
100 W, 20 μs	100 W, 40 μs	100 W, 60 μs	100 W, 80 μs
120 W, 20 μs	120 W, 40 μs	120 W, 60 μs	120 W, 80 μs
160 W, 20 μs	160 W, 40 μs	160 W, 60 μs	160 W, 80 μs
200 W, 20 μs	200 W, 40 μs	200 W, 60 μs	200 W, 80 μs

**Table 2:** Summary of the SLM parameters in building 3D specimens, (green) nearly full amorphous structure, (yellow) partially crystallized.

Laser Parameters				
100 W, 30 μs	100 W, 40 μs	100 W, 60 μs	100 W, 80 μs	100 W, 100 μs
120 W, 30 μs	120 W, 40 μs	120 W, 60 μs	120 W, 80 μs	120 W, 100 μs
160 W, 30 μs	160 W, 40 μs	160 W, 60 μs	160 W, 80 μs	160 W, 100 μs
180 W, 20 μs	180 W, 30 μs	180 W, 40 μs	180 W, 50 μs	180 W, 60 μs
200 W, 20 μs	200 W, 30 μs	200 W, 40 μs	200 W, 50 μs	200 W, 60 μs

**Table 3:** Crystallization enthalpy and volume fraction of the amorphous phase of as-cast and SLM specimens with parameters of P = 200 W, t = 40, 50, 60, and 70 μs.

	As-Cast	200 W, 40 μs	200 W, 50 μs	200 W, 60 μs	200 W, 70 μs
△H (J/g)	−49.2	−47.7	−40.0	−29.3	−22.7
$V_{amorphous}$	-	96.95%	81.3%	59.55%	46.14%

**Table 4:** Corrosion potential and corrosion current density of as-cast specimen and SLM specimens with parameters of P = 200 W, t = 40, 50, 60, and 70 μs.

	As-Cast	200 W, 40 μs	200 W, 50 μs	200 W, 60 μs	200 W, 70 μs
Ecorro (V)	−0.373	−0.370	−0.366	−0.359	−0.351
Icorro (A)	−9.77e^−8^	−1.65e^−7^	−2.79e^−7^	−6.79e^−7^	−1.87e^−6^
CPR (μm)	0.11	0.18	0.31	0.76	2.09
